# Effects of Warming Temperatures on Winning Times in the Boston Marathon

**DOI:** 10.1371/journal.pone.0043579

**Published:** 2012-09-26

**Authors:** Abraham J. Miller-Rushing, Richard B. Primack, Nathan Phillips, Robert K. Kaufmann

**Affiliations:** 1 Department of Biology, Boston University, Boston, Massachusetts, United States of America; 2 Department of Geography and Environment, Boston University, Boston, Massachusetts, United States of America; University of Zaragoza, Spain

## Abstract

It is not known whether global warming will affect winning times in endurance events, and counterbalance improvements in race performances that have occurred over the past century. We examined a time series (1933–2004) from the Boston Marathon to test for an effect of warming on winning times by men and women. We found that warmer temperatures and headwinds on the day of the race slow winning times. However, 1.6°C warming in annual temperatures in Boston between 1933 and 2004 did not consistently slow winning times because of high variability in temperatures on race day. Starting times for the race changed to earlier in the day beginning in 2006, making it difficult to anticipate effects of future warming on winning times. However, our models indicate that if race starting times had not changed and average race day temperatures had warmed by 0.058°C/yr, a high-end estimate, we would have had a 95% chance of detecting a consistent slowing of winning marathon times by 2100. If average race day temperatures had warmed by 0.028°C/yr, a mid-range estimate, we would have had a 64% chance of detecting a consistent slowing of winning times by 2100.

## Introduction

Winning times in endurance events such as marathons have improved over the last century [1], a trend that may slow or reverse should temperatures warm as climate models predict [2]. Previous studies have shown that warm temperatures diminish the performance of endurance athletes by affecting their ability to regulate internal body temperature [3], [4], [5], [6], [7], [8]. However, it is not known whether warming trends have already affected athletes' performance during endurance competitions, or if they will affect athletic performance over the next century.

The potential for warming temperatures to affect marathon athletes depends on the magnitude of warming and interannual variation in daily temperatures. If temperatures do not increase relative to temperature variability on race days, the effects of warming on marathon times may not be detectable. However, at some point temperature increases may be large enough to affect marathon times.

The Boston Marathon provides an ideal case to test for an effect of warming on marathon times. It is the oldest continuous annual marathon in the world. It has been run on the same course on approximately the same day each year since 1924. Its consistent route and the length of the recorded time series are exceptional. Thus, we used the Boston Marathon to test whether warming has affected the winning times for both men and women endurance runners.

## Materials and Methods

### Weather and race data

We compiled weather data for 1933–2004 from the Blue Hill Meteorological Observatory. Weather data were incomplete for years prior to 1933. The station is located in Milton, Massachusetts, approximately 9 km south of Boston, and has the longest continuous weather record in the United States [9]. Weather observations at this station are strongly correlated to observations at other weather stations in the Boston area (data not shown), and offer the most complete weather record available, although records are missing for 1962–1964.

We used weather observations made between 12:00 pm and 2:00 pm on the day of the marathon. Through 2005, the race started at 12:00 pm. The tight correspondence between the time of weather observations and the race time limited the impact of weather variability on race day itself on the analysis. The race was run on April 19 until 1969, when it changed to the third Monday in April (dates ranged from April 15 to April 21).

**Figure 1:**
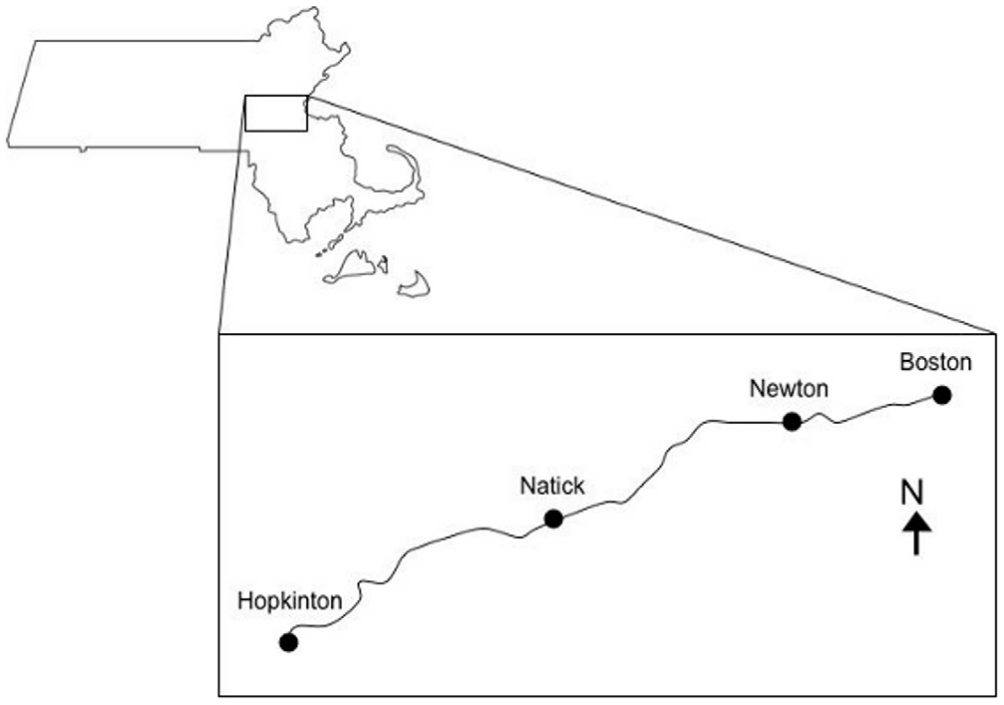
Map of the course of the Boston Marathon. The course runs 42.2 km from Hopkinton to Boston, Massachusetts.

Weather data included temperature, wind direction, wind speed, humidity, wind chill, sky cover, and precipitation. For simplicity, we converted wind direction and speed into a single variable that included information on headwinds and tailwinds. We divided wind into two component vectors: one parallel to the racecourse, which was approximately SW to NE (Figure 1), and one perpendicular to the course. We included only the component parallel to the course in our analysis because it had the greatest potential to affect running speed. We assigned tailwinds (SW) positive values and headwinds (NE) negative values.

In addition to the weather variables considered here, wet bulb globe temperature is often used as an index of heat stress for athletes [10], [11]. Wet bulb globe temperature, a single value, provides information about temperature, humidity, and solar radiation (sky cover). Instead of considering wet bulb temperature in our model, we considered each factor separately because we wanted to isolate the role of temperature. By separating temperature, humidity, and solar radiation, we were able to examine how warming might affect winning times in marathons.

We analyzed men's winning times starting in 1933, the earliest year with complete weather data, and ending in 2004. We analyzed women's winning times from 1972 to 2004. Women first officially ran in the Boston Marathon in 1972. Beginning in 2006, the starting times for the race were moved to earlier in the day, making it difficult to distinguish between the effects of warming temperatures across years and the effects of starting at a cooler time of day. According to a press release put out by the Boston Athletic Association, the change in time was made to provide cooler conditions for the race, allow for earlier re-opening of roads to vehicular traffic, address runner preferences for a morning start time, and support emergency support staff.

### Analysis

**Figure 2:**
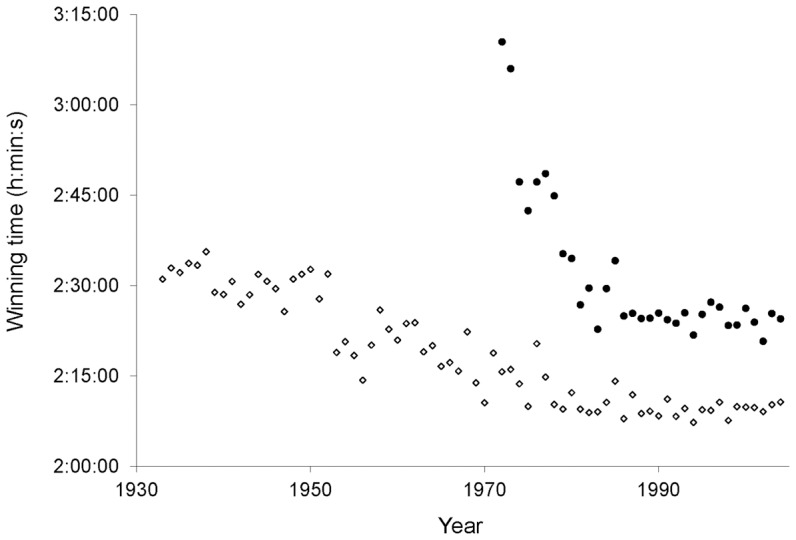
Winning marathon times over the history of the Boston Marathon. Open diamonds represent men's times from 1933–2004. Closed circles represent women's times from 1972–2004. Women's running times improved rapidly in the first 14 yr of women's participation in the marathon. From 1983 to 2004, the differences between men and women's winning times held relatively constant at an average of 15 min 47 s.

We used multiple regression to determine the relationships between weather variables and the winning times for men and women runners. Winning times for both men and women generally improved over time (Figure 2). To account for this trend, we specified world record marathon times as an explanatory variable in the multiple regression model. Regression errors of the relationships between winning times in the Boston Marathon and the world record times did not contain any trends over time (men, *P* = 0.40; women, *P* = 0.64), nor did they contain unit roots as determined by an augmented Dickey–Fuller test [12].

We used Monte Carlo techniques to estimate the power to detect warming on race day in the future. We used the following equation to generate one thousand experimental data sets for race day weather, each with temperature observations from 1933 to 2100:
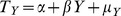
in which *T* was temperature in year *Y* of the experimental data set, 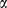 was a constant, 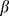 represented the linearized annual rate of warming, and *µ* was an error term. The error term was drawn randomly from a normal distribution with a mean of zero and a standard deviation of 6.3, which was the standard deviation of race day temperatures 1933–2004. This procedure assumed that April daily temperature variability will remain constant in the future. This model also omitted the potential influence of difficult-to-project factors, such as prize money and competitiveness.

We used this technique to generate three experimental data sets of temperatures from 1933 to 2100, each data set representing a different warming scenario. In all three data sets, we set 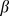 equal to 0.017°C/yr for the years 1933 to 2005. This rate of warming was the rate at which mean maximum April temperatures increased from 1893 to 2005 as determined by simple linear regression (*P*<0.001). We did not have mean maximum April temperature data from years prior to 1893. We used the entire observational temperature record available to us at this site to estimate the rate of warming and variability in daily temperatures (see next paragraph) for our experimental data sets. The first experimental data set maintained the historical rate of warming (0.017°C/yr) until 2100. The second and third data sets simulated two warming scenarios–0.028°C/yr and 0.058°C/yr warming from 2006 to 2100–consistent with mid- and high-range estimates for seasonal warming in this region [2], [13], [14].

Although we assumed that the magnitude of the long-term trend in warming temperatures on race day would match the magnitude of change in seasonal temperatures, we imposed the observed historical variation in temperatures on race day on our simulation. The variation in daily temperatures (sd = 6.3) was an order of magnitude larger than the variation in annual temperatures (sd = 0.65).

We then used each experimental temperature data set to test whether we could detect a slowing of winning times over time. For each experimental data set, we calculated winning times as:
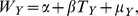
where *W* was the winning time in year *Y*, 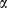 was a constant, and *µ* was an error term. We calculated 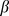, the linearized effect of temperature (*T*) on winning times, from our multiple regression model that described the effect of weather on winning times from 1933–2004 for men and 1972–2004 for women (Table 1). We held all other weather variables constant and thus omitted them from this equation.

**Table 1:** Regression results showing effects of temperature and wind on winning times in the Boston Marathon.

Explanatory variable	Men		Women	
	Coefficient	*P*	Coefficient	*P*
World record	1.00	<0.001	1.37	<0.001
Temperature	20.29	<0.001	21.07	0.015
Wind	−21.19	<0.001	−25.06	0.024

The units for the regression coefficients are s/°C for temperature and s/(m/s) for wind. The coefficients for world records are unitless because both world record times and winning times in the Boston Marathon had the same units. For wind, we assigned tailwinds positive values and headwinds negative values.

Finally, we used simple linear regression to test whether we could detect a significant increase (*P*<0.05) in winning times for five consecutive years for each experimental data set. We tested for changes in winning times from 1933 for men or 1972 for women through the year 2100. The requirement to find a significant trend for five consecutive years prevented an anomalously warm year from causing a significant change in winning times.

## Results

The complete regression model relating winning times in the Boston Marathon to weather included three explanatory variables: world record times, wind, and temperature. We did not include humidity, sky cover, wind chill, and precipitation because they did not have a statistically significant effect on winning times (*P*>0.05). Although Trapasso and Cooper [3] found that humidity and sky cover affected running times, we, like two other studies [4], [15], found no such effects in our study.

**Figure 3:**
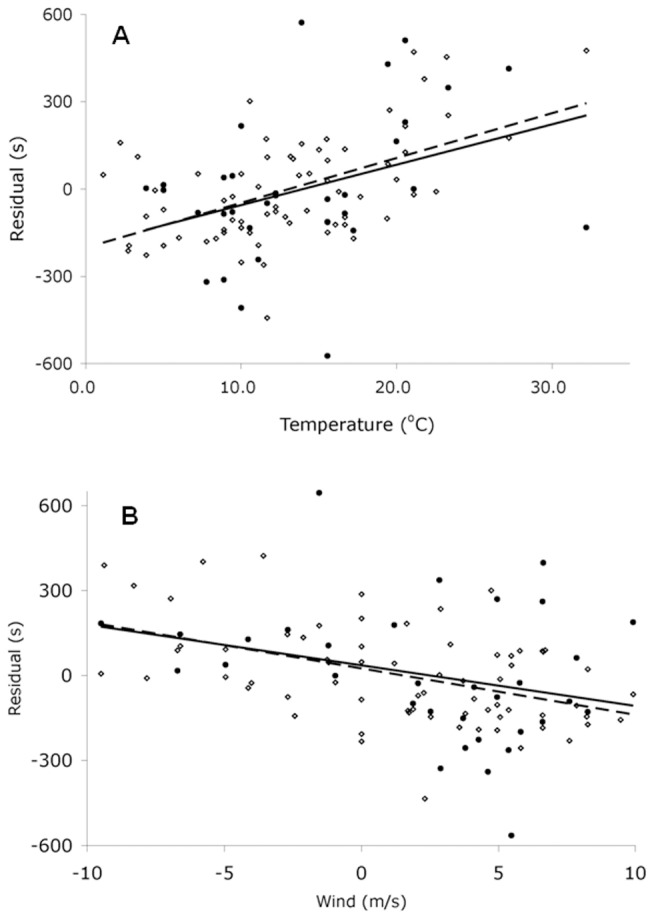
Effects of (A) temperature and (B) wind on winning times in the Boston Marathon from 1933 to 2004. Using coefficients determined by least squares regression (Table 1), we estimated winning times from the equations, (A) predicted winning time  = 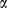 + 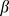
_1_world record + 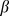
_2_wind and (B) predicted winning time  = 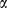 + 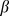
_1_world record + 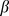
_2_temperature. The residuals plotted here represent the difference between each year's predicted winning time and actual winning time (residual  =  actual – predicted). Open diamonds represent men's residuals. Solid circles represent women's residuals. Lines represent least squares best fit for men (dashed) and women (solid).

Results indicated that a 1°C increase in temperature slowed the winning time for men by 20 s (se = 3.3 s, *P*<0.001), and 21 s (se = 8.2 s, *P* = 0.015) for women (Figure 3a, Table 1). Men set 11 of 16 (69%) course records at temperatures below 13.1°C, the median race temperature for men's races (1933–2004), and 6 of 16 (38%) records at temperatures below 8.9°C, the lowest quartile, frequencies no different than would be expected by chance (median, χ^2^ *= *2.25, *P* = 0.134; quartiles, χ^2^ = 3.50, *P* = 0.321). Women set 6 of 9 (67%) course records on days below 12.2°C, the median race temperature for women's races (1972–2004), and 3 of 9 (33%) records at temperatures below 8.9°C, the lowest quartile, also frequencies no different than would be expected by chance (median χ^2^ *= *1.00, *P* = 0.317; quartiles, χ^2^ *= *1.22, *P* = 0.748). Headwinds slowed the winning time for men by 21 s per 1 m/s wind (se = 4.2 s, *P*<0.001), while women's times slowed 25 s per 1 m/s wind (se = 10.6 s, *P* = 0.024) (Figure 3b, Table 1); tailwinds had the opposite effect. Men experienced tailwinds above 4 m/s during 38% of races (1933–2004), while women experienced tailwinds of more than 4 m/s during 48% of races (1972–2004). Men set 56% of course records during tailwinds above 4 m/s and women set 67%, a frequency no different than would be expected by chance (men, χ^2^ *= *2.69, *P* = 0.260; women χ^2^ *= *2.00, *P* = 0.368). Overall, temperature and wind conditions explained 35% of the variation in men's winning times and 17% of variation in women's winning times, as shown by partial *R* ^2^, after we accounted for improvement in winning times over time (using changes in world record times). Race-time temperatures ranged from 1.1 to 32.2°C and wind speeds ranged from a headwind of 9.4 m/s to a tailwind of 9.9 m/s.

Between 1893 and 2005, Boston mean annual temperatures warmed by an average of 0.015°C/yr (*P*<0.001), more than double the global average [2]. Mean maximum April temperatures in Boston warmed an average of 0.017°C/yr over the same time period (*P*<0.001). These large increases in Boston temperature are due in part to the urban heat island effect [14], [16]. Nonetheless, we cannot detect a trend in race day temperatures since 1933 (*P* = 0.76) because of high interannual variation (range 1.1–32.2°C, mean  = 13.4°C, median  = 13.1°C, standard deviation  = 6.3°C). Thus, warming did not have a detectable effect on the winning times of the Boston Marathon between 1933 and 2004.

To determine whether warming might have been expected to have a detectable effect on marathon times by the year 2100 had the starting time remained consistent, we used experimental data sets generated by Monte Carlo techniques to simulate the effect of sustained warming at historical rates (0.017°C/yr) and two warming scenarios: 0.028°C/yr, and 0.058°C/yr warming over the next 100 yr. On average, these warming scenarios would slow marathon times between 34 s (0.017°C/yr warming for men) and 2 min 4 s (0.058°C/yr warming for women) over the next 100 yr. We used simulated race day temperatures to calculate the winning times for men and women marathon runners for each year and fit a time trend to the observations for each of the 1,000 experimental data sets.

**Figure 4:**
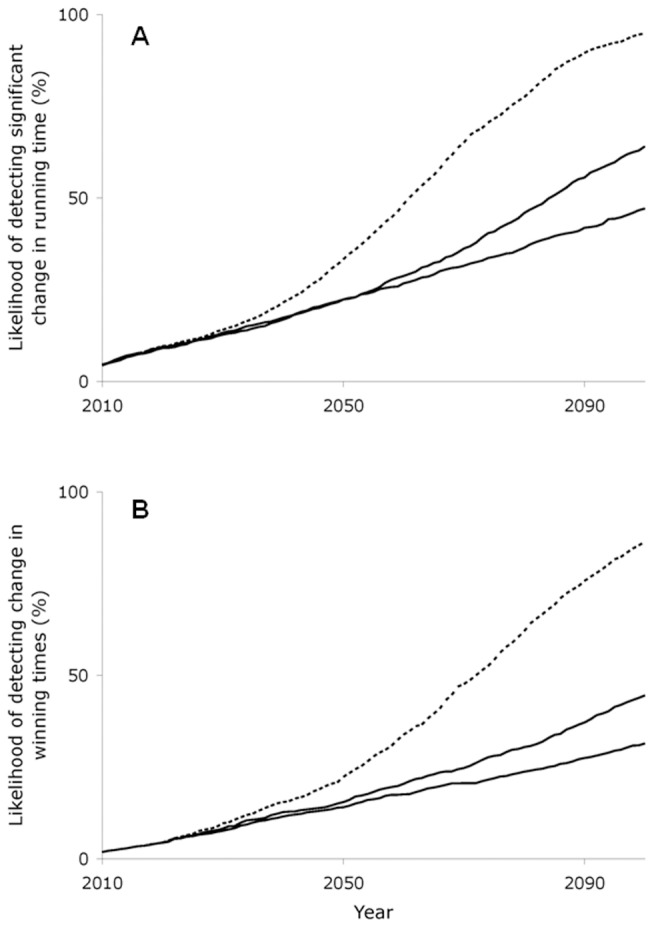
Likelihood of detecting a change in winning times for (A) men and (B) women. Lines show percent of experimental data sets (based on 1000 trials) that showed a significant change (*P*<0.05) in winning times for five consecutive years for each warming scenario. Each experimental data set began with 0.017°C/yr warming until 2006. After 2006, the each warming scenario maintained a different rate of warming until 2100: 0.017°C/yr (solid lines), 0.028°C/yr (dashed lines), and 0.058°C/yr (dotted lines). Men's data started in 1933. Women's data began in 1972.

For men (starting in 1933), the percentage of experimental data sets in which the coefficient associated with the time trend was statistically different from zero for five consecutive years by the year 2050 ranged from 22% for 0.017°C/yr and 0.028°C/yr warming to 33% for 0.058°C/yr warming (Figure 4a). We could detect a significant change in winning times by 2100 in 47% of cases with 0.017°C/yr warming, 64% with 0.028°C/yr, and 95% with 0.058°C/yr. For women (starting in 1972), the percentage of experimental data sets in which the coefficient associated with the time trend was statistically different from zero for five consecutive years by the year 2050 ranged from 14% for 0.017°C/yr warming to 15% for 0.028°C/yr and 22% for 0.058°C/yr (Figure 4b). We could detect a significant change in winning times by 2100 in 31% of cases with 0.017°C/yr warming, 45% with 0.028°C/yr, and 86% with 0.058°C/yr. That is, by 2100, had starting times remained constant over time, we would have had a 95% chance of detecting a slow down in men's winning times, and a 86% chance for women's winning times given the highest predicted temperature increase associated with global climate change (0.058°C/yr).

## Discussion

Previous studies have shown that relatively modest increases in race-day temperatures can slow marathon performances [6], [7], [8]. However, despite significant warming in Boston since 1933, large variation in daily temperatures (an order of magnitude larger than variation in annual temperatures) has masked any changes in winning times associated with long-term changes in temperatures on race day. If temperatures warm as much as is predicted by the high end of IPCC estimates [2], which is reasonable to expect in Boston [13], [14], warming would likely have a detectable effect on marathon times by the year 2100 if starting times remained consistent over time (Figure 4). Even if temperatures warm at the mid-range rate of 0.028°C/yr, we would have a 64% chance of detecting an effect of warming on Boston Marathon times by the year 2100. By changing starting times for the race to earlier in the day when temperatures are cooler, however, race organizers have effectively counteracted any effects that long-term warming would have had on winning times. If this change had not been made, we would have expected that warming would likely lead to fewer record-breaking times in the Boston Marathon.

Our results also show that the effect of temperature on winning marathon times for men and women are strikingly similar (Table 1). For both sexes, winners ran fastest at relatively cold temperatures, as has been found previously [6], [7]. The similarity between the sexes' responses to warming temperatures suggests that warming temperatures may not affect the difference between men's and women's winning times. This difference has remained relatively steady in the Boston Marathon (Figure 2) and other endurance races [17], [18] since the mid-1980 s.

Notably, there was one strong outlier runner, who ran a particularly fast time given the race conditions. In 1976, race day temperatures reached 32.2°C, the hottest Boston Marathon on record. The race was slow for the men, but was surprisingly fast for the women (Figure 3a); it is possible that the dramatic yearly improvements in women's long-distance running times during the 1970 s exerted a greater influence than the weather. The winning woman finished 2 min 31 s faster than the model predicted. In 2004, another hot year, race day temperatures reached 27.2°C. In that year the race was predictably slow after accounting for a strong tailwind (Figure 3a).

Previous studies have shown that other weather variables, such as humidity, precipitation, and cloud cover, can also affect the winning times in marathons [3], [6]. We did not find these effects in our study. However, each of these variables clearly affect endurance athletes in many circumstances, as these variables are known to alter the ability of athletes to regulate their internal body temperatures [5]. Runners transfer heat away from the body through radiation, convection, and sweat [5]. Warmer air temperatures narrow the skin-to-air temperature gradient and reduce dry heat loss. High relative humidity decreases the rate at which sweat evaporates. Direct solar radiation, as experienced on days with clear skies, increases radiative heat gain [19].

Moreover, the relative importance of different weather factors can change as temperatures change. For example, relative humidity and wet bulb temperature are known to become increasingly important for performance as air temperatures increase [10]. Thus, relative humidity may exert more influence on winning times in the Boston Marathon as temperatures warm in the future. Additionally, the effects of particular weather conditions on marathon winners can vary depending on the location of the race and the time period studied, as is suggested by our results in combination with those of previous studies [3], [4], [6], [8]. For example, we found that wind had a significant effect on winning times in the Boston Marathon (Table 1, Figure 3b). That effect may be caused by the largely straight-line route of the Boston Marathon. Wind may not have such a strong effect on marathons with a more circular route.

In summary, despite the well-known effect of temperature on marathon performance 3,4,5,6,7,8], we found that warming trends in Boston have not caused winning times to slow over time because of high variability in temperatures on race day. However, our models indicate that if race starting times had not changed and average race day temperatures continue to warm by 0.058°C/yr, a high-end estimate, we would have had a 95% chance of detecting a consistent slowing of winning marathon times by 2100. If average race day temperatures warm by 0.028°C/yr, a mid-range estimate, we would have had a 64% chance of detecting a consistent slowing of winning times by 2100.

## References

[1] Whipp BJ, Ward SA (1992) Will women soon outrun men? Nature 355: 25. 1731197 10.1038/355025a0

[2] IPCC (2007) Climate Change 2007: The Physical Science Basis. Contribution of Working Group I to the Fourth Assessment Report of the Intergovernmental Panel on Climate Change. Cambridge: Cambridge University Press. 996 p.

[3] Trapasso LM, Cooper JD (1989) Record performances at the Boston Marathon: Biometeorological factors. International Journal of Biometeorology 33: 233–237. 2613367 10.1007/BF01051083

[4] Zhang SP, Meng GL, Wang YW, Li J (1992) Study of the relationships between weather conditions and the marathon race, and of meteorotropic effects on distance runners. International Journal of Biometeorology 36: 63–68. 1634282 10.1007/BF01208915

[5] Cheuvront SN, Haymes EM (2001) Thermoregulation and marathon running. Sports Medicine 31: 743–762. 11547895 10.2165/00007256-200131100-00004

[6] Ely MR, Cheuvront SN, Roberts WO, Montain SJ (2007) Impact of weather on marathon-running performance. Medicine and Science in Sports and Exercise 39: 487–493. 17473775 10.1249/mss.0b013e31802d3aba

[7] El Helou N, Tafflet M, Berthelot G, Tolaini J, Marc A, et al (2012) Impact of environmental parameters on marathon running performance. PloS ONE 7: e37407. 22649525 10.1371/journal.pone.0037407 PMC3359364

[8] Martin DE, Buoncristiani JF (1999) The effects of temperature on marathon runners' performance. Chance 12: 20–25.

[9] Conover JH (1985) Highlights of the history of the Blue Hill Observatory and the early days of the American Meteorological Society. Bulletin of the American Meteorological Society 66: 30–37.

[10] Yaglou CP, Minard D (1957) Control of heat casualties at military training centers. AMA Archives of Industrial Health 16: 302–316. 13457450

[11] McCann DJ, Adams WC (1997) Wet bulb globe temperature index and performance in competitive distance runners. Medicine and Science in Sports and Exercise 29: 955–961. 9243496 10.1097/00005768-199707000-00016

[12] Dickey DA, Fuller WA (1979) Distribution of the estimators for autoregressive time series with a unit root. Journal of the American Statistical Association 74: 427–431.

[13] Hayhoe K, Wake C, Huntington T, Luo L, Schwartz M, et al (2006) Past and future changes in climate and hydrological indicators in the US Northeast. Climate Dynamics 28: 381–407.

[14] New England Regional Assessment Group (2001) Preparing for a changing climate: The potential consequences of climate variability and change. New England regional overview. Durham, NH: University of New Hampshire, U.S. Global Change Research Program. 96 p.

[15] Ely MR, Cheuvront SN, Montain SJ (2007) Neither cloud cover nor low solar loads are associated with fast marathon performance. Medicine and Science in Sports and Exercise 39: 2029–2035. 17986912 10.1249/mss.0b013e318149f2c3

[16] Zhang XY, Friedl MA, Schaaf CB, Strahler AH, Schneider A (2004) The footprint of urban climates on vegetation phenology. Geophysical Research Letters 31: doi:10.1029/2004GL020137.

[17] Sparling PB, O'Donnell EM, Snow TK (1998) The gender difference in distance running performance has plateaued: An analysis of world rankings from 1980 to 1996. Medicine and Science in Sports and Exercise 30: 1725–1729. 9861606 10.1097/00005768-199812000-00011

[18] Cheuvront SN, Carter III R, DeRuisseau KC, Moffatt RJ (2005) Running performance differences between men and women: An update. Sports Medicine 35: 1017–1024. 16336006 10.2165/00007256-200535120-00002

[19] Nielsen B, Kassow K, Aschengreen FE (1988) Heat balance during exercise in the sun. European Journal of Applied Physiology 58: 189–196. 10.1007/BF00636625 3203666

